# Proliferative effects of nanobubbles on fibroblasts

**DOI:** 10.1007/s13534-022-00242-y

**Published:** 2022-08-13

**Authors:** Hansol Heo, Junseon Park, Jeong II Lee, Jungho Kim, Joong Yull Park, Jong-Min Kim

**Affiliations:** 1 School of Mechanical Engineering, Chung-Ang University, Seoul, 06974 Republic of Korea; 2 Department of Computer Science and Engineering, Chung-Ang University, Seoul, 156-756 Republic of Korea; 3 Department of Life Science, Sogang University, Seoul, 04107 Republic of Korea; 4 Department of Intelligent Energy and Industry, Chung-Ang University, Seoul, 06974 Republic of Korea

**Keywords:** Nanobubble, Gas–liquid mixing, Cell cycle, Cell proliferation

## Abstract

In recent years, the potential of nanobubbles (NBs) for biological activation has been actively investigated. In this study, we investigated the proliferative effects of nitrogen NBs (N-NBs) on fibroblast cells using cell assays with image analysis and flow cytometry. A high concentration of N-NBs (more than 4 × 10^8^ NBs/mL) was generated in Dulbecco’s modified Eagle’s medium (DMEM) using a gas–liquid mixing method. In image analysis, the cells were counted and compared, which showed an 11% increase in cell number in the culture medium with N-NBs. However, in two further cell cytometry analyses, the effect of nanobubbles on cell division was found to be insignificant (approximately 2%); as there is insufficient evidence that N-NB is involved in cell division mechanism, further studies are needed to determine whether NB affects other cellular mechanisms such as apoptosis. This study presents the first successful attempt of directly generating and quantifying N-NBs in a culture medium for cell culture. The findings suggest that the N-NBs in the culture medium can potentially facilitate cell proliferation.

**Supplementary Information:**

The online version contains supplementary material available at 10.1007/s13534-022-00242-y.

## Introduction

Cells are controlled by a variety of stimuli such as biological growth factors, hormones, electrical fields, and mechanical forces [1]. Proliferation is a factor affected by external stimulation, which can be easily and quickly observed and analyzed. In tissue engineering and regenerative medicine, the enhancement of cell proliferation is of great significance. Various stimuli including not only biological factors but also engineered conditions have been investigated to improve cell proliferation. Hypoxic conditions and fibroblast growth factor-2 improved the proliferation of human bone marrow stromal cells [2]. Gelatin microparticles loaded with ephedra extract improved the proliferation of human lung epithelial cells [3]. The mouse fibroblast cell line L929 was found to be positively responsive to electric stimulation in terms of cell division [4]. Recently, nanobubbles (NBs) are being investigated for their potential application to improve cell proliferation. NBs refer to gas-filled cavities less than 1 µm in size in liquid. From the perspective of classical thermodynamics, NBs should diffuse and disappear in water within the order of microseconds due to high internal pressure [5, 6]; however, various studies have reported results (theoretical and experimental approaches) demonstrating that NBs can exist in a stable state [7, 8]. Therefore, NBs can be maintained in liquid for a long time because the floating speed of NBs is negligible due to their small size. NBs have some unique properties. As NBs present in liquid have a high specific surface area due to their small size, their solubility may be markedly higher compared with that of micro- or macro-sized bubbles [9]. Highly reactive free radicals generated from thermal decomposition caused by bubble collapse during the NB generation process are adsorbed to the bubble interface, contributing to the electrical properties of NBs [10]. In addition, NBs have been reported to increase the mobility of water molecules based on nuclear magnetic resonance (NMR) relaxation time measurements [11]. Due to their unique physicochemical properties, NBs have attracted considerable attention in several areas [12–15], including biomedical applications [16, 17]. Recently, several studies have been conducted on the biological effect of NBs as well as nano-materials on living organisms [11, 18–23]. The overall effect of NBs on the growth of plants, fishes, and mice was investigated in 2013, which revealed that NBs accelerated the growth of plants, fishes, and mice and increased their (fishes and mice) weight [15]. Although the mechanism of NBs in growth promotion remains unclear, NBs are known to play an important role in accelerating the growth of living organisms. More recently, the effect of NBs was assessed by comparing seed germination in NB water with seed germination in distilled water and H_2_O_2_ solutions through the measurement of superoxide radicals (O_2_^•−^) in seeds by nitroblue tetrazolium (NBT) staining [20]. The levels of superoxide radicals in NB water and 0.3 mM H_2_O_2_ solution were similar and considerably higher than in distilled water. The production of reactive oxygen species (ROS, ^•^OH) by NBs might contribute to the physiological induction of seed germination. In addition, NBs adsorbed on polystyrene films were used as a scaffold for the cell culture of mouse fibroblast L929 cells to show that the NBs could promote the proliferation of fibroblast cells [24]. The findings demonstrated the positive effect of NBs on plants, animals, and cells. However, some other studies have also reported the suppressive/negative effect of NBs. Therefore, NBs may or may not have a positive effect depending on the given conditions, and each case should be investigated in detail to understand the mechanism of NBs. In a previous study, the proliferation of dental follicle stem cells in a culture medium containing air and oxygen NBs was inhibited due to the high oxygen content of the NBs [25]. The above-mentioned studies suggest that NBs may have different (positive and negative) effects on biological activation depending on the type of organism or type of gas in the NBs. Nevertheless, there are limited studies on cell proliferation using a cell medium containing NBs.

In this study, we propose a new cell culture method using nitrogen NBs (N-NBs) and investigated the possible effect of NBs on cell (fibroblast) proliferation. We used nitrogen gas, which is the most abundant gas in the atmosphere and has low reactivity, in this work because we wanted to evaluate the effect of NBs itself and not of the gas types such as oxygen and hydrogen. The N-NBs were directly generated in Dulbecco’s modified Eagle’s medium (DMEM), and Medical Research Council cell strain 5 (MRC-5) cells were cultured with DMEM containing N-NBs, which are fibroblast cells. Fibroblasts are the most common cells of the connective tissue and play a major role in intercellular bonding and wound healing. In addition, cell culture protocols are universal, making them suitable for evaluating external gaseous stimuli that might affect proliferation. For NB generation, a gas–liquid mixing method was used, and the concentration and size of the N-NBs were examined. We also performed image analysis and flow cytometry to evaluate cell proliferation and demonstrated that cell proliferation in the culture medium with N-NBs was enhanced. To the best of our knowledge, this study is the first to generate NBs directly in a culture medium (DMEM), which may be an efficient method to investigate the effect of NBs on cells. Previously, the NB solution is typically prepared separately and mixed with the culture medium prior to cell culture experiments. The findings of this study could be used to expand the scope of application for various cell types and gaseous NBs and could contribute to further research on the biological applications of NBs in the future.

## Materials and methods

### Materials

For cell culture, MRC-5, which is a fibroblast cell line, was purchased from the Korean Cell Line Bank (Korean Cell Line Research Foundation, Republic of Korea). DMEM (Gibco®, Thermo Fisher Scientific, USA) was used as a basal medium to culture MRC-5 cells. Fetal bovine serum (FBS; Gibco®, Thermo Fisher Scientific, USA) and Antibiotic–Antimycotic (Anti-Anti, 100X; Gibco®, Thermo Fisher Scientific, USA) were used as supplements. For NB generation in DMEM, highly-purity nitrogen gas (99.999%; Shinyoung Gas Co., Republic of Korea) was used.

### Preparation of cell medium with N-NBs

In this study, a gas–liquid mixing (agitation) method with a linear actuator was used [26] to generate a large number of N-NBs in DMEM in a short time. A sterilized disposable conical tube was dipped in DMEM vertically, and nitrogen gas was supplied to fill half of the tube. The tube was then capped and sealed with parafilm to prevent the influx of contaminants such as particles, oil, and microbes, which can negatively affect the cell culture. The sealed tube was mounted onto an actuator and agitated with the set cycle (117 strokes/min). After N-NB generation in DMEM, the cell medium with N-NBs was prepared by mixing the DMEM containing N-NBs with 10% FBS and 1% Anti-Anti.

### Measurements of concentration and size of N-NBs in DMEM

For the analysis of N-NBs in DMEM, a nanoparticle tracking analysis (NTA) method was used with a NTA instrument (NanoSight LM10-HSBFT14; Malvern, UK), which is widely used in the NB research field [27, 28]. It is a NB visualization technique that provides size, count and concentration measurements. The NTA instrument was equipped with a charge-coupled device (CCD) camera to capture the dispersed light and a red laser light source with a wavelength of 642 nm to excite fluorescence. The DMEM containing N-NBs was placed in the sample chamber, which had a volume of 0.3 mL. With laser light illumination, the N-NBs appeared individually as fast-moving dispersed dots of light (white dots) under Brownian motion, which were automatically tracked and captured by the CCD camera. Subsequently, the NTA image analysis program (i.e., the NTA software) determined the concentration and size of the N-NBs in DMEM. In addition, changes in the concentration and size of the N-NBs in DMEM was observed for 48 h to ensure the presence and stability of the N-NBs during the cell culture process.

### Cell culture experiment

We used MRC-5 cells between passage 10 and 15 in the experiment. The cells were cultured in an incubator at 37 °C with 5% CO_2_ and 95% humidity using a culture medium containing DMEM supplemented with 10% FBS and 1% Anti-Anti. There was a slight difference in the cell culture process due to the difference in the analysis method as follows.

First, cell proliferation was analyzed by image analysis. To obtain reliable data, cell culture experiments were performed 5 times independently. To attach the cells, we loaded 1 mL of non-NB culture medium including MRC-5 cells (5 × 10^3^ / mL) in 24-well plates. After 24 h of cell attachment, NucBlue® Live ReadyProbes™ reagent (NucBlue; Invitrogen™, Thermo Fisher Scientific, USA) was added (1 drop per well) and incubated at 37 °C for 30–40 min to stain the cells. Images at the center of the well were taken using an inverted fluorescence microscope (CKX41; Olympus, Japan). Then, half of the 24 well was replaced with the culture medium with N-NBs (experimental group), and the remaining half of the 24well was replaced with the fresh culture medium without N-NBs (control group). After incubation for 48 h, NucBlue staining was performed, and images of the cells were taken. To count the number of cells per image, image analysis was performed using imageJ. (US National Institutes of Health, USA).

Second, cell proliferation was analyzed by flowcytometry. The cells were stained using CellTrace™ carboxyfluorescein succinimidyl ester (CFSE) Cell Proliferation Kit (Invitrogen™, Thermo Fisher Scientific, USA). CFSE dye was diluted in dimethyl sulfoxide (DMSO) at a concentration of 5 mM to make a stock solution, and the stock solution was diluted in PBS at a ratio of 1000:1 to prepare a PBS-dye solution. The cells were stained by adding 1 mL of the PBS-dye solution to 1 × 10^6^ cells in suspension and incubated at 37 °C for 20 min. After adding the culture medium without N-NBs to the stained cell solution (5 ×) with incubation for 5 min, the cell was pelleted using centrifuge, and the supernatant was removed. The stained cells were seeded in *Φ*150-culture dishes (1 × 10^6^ cells each). After 24 h of cell attachment in culture medium without N-NBs, half of culture dishes was replaced with the culture medium with N-NBs (experimental group), and the remaining half of culture dishes was replaced with the fresh culture medium without N-NBs (control group), followed by incubation for 48 h. Subsequently, the cells were detached from the dish using trypsin (Gibco®, Thermo Fisher Scientific, USA), and flow cytometry analysis was performed using FACS Canto™II (Becton, Dickinson and Company, USA).

## Results and discussion

### NB generation in DMEM

**Fig. 1:**
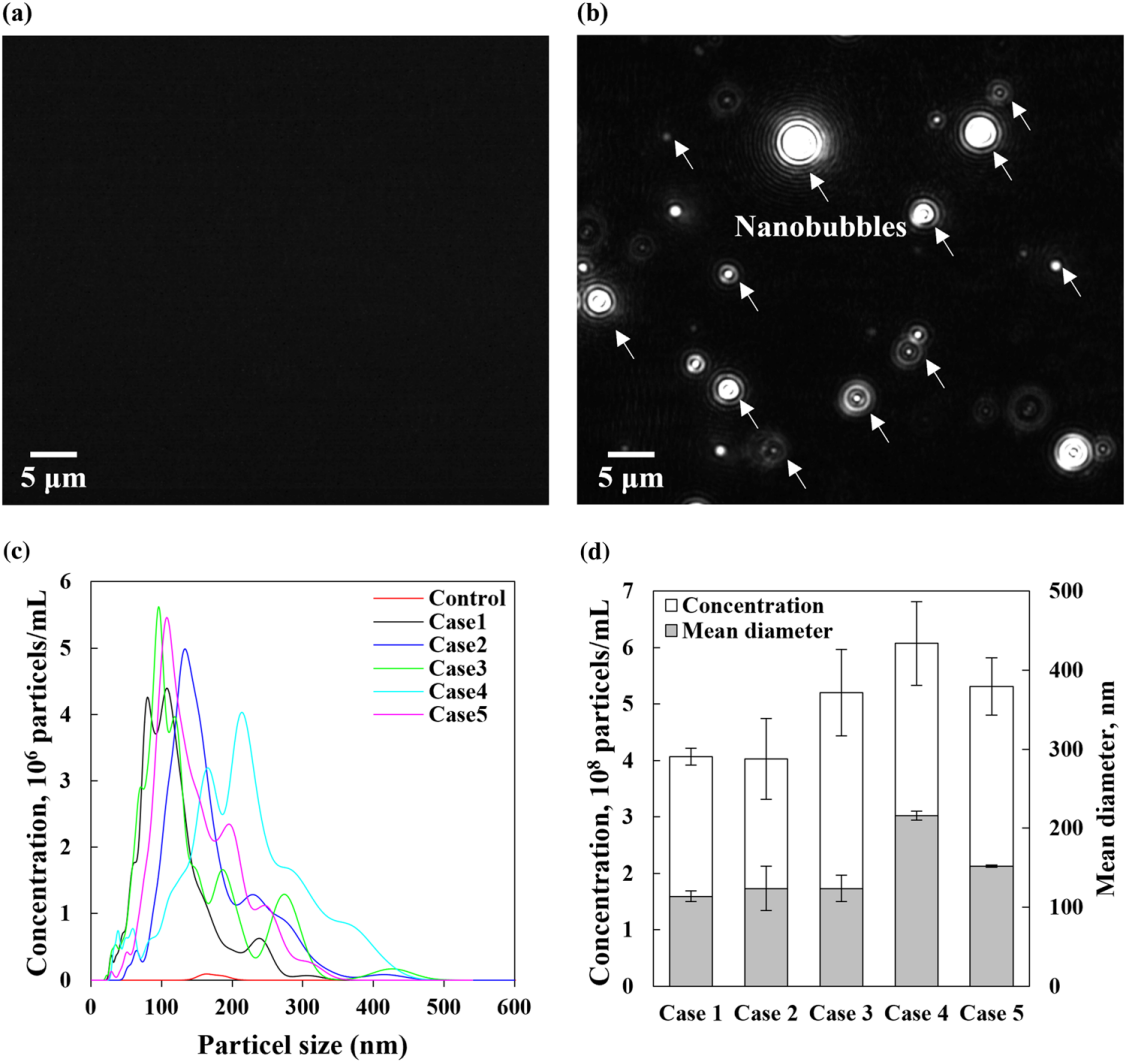
Results of NB analysis performed using the NTA apparatus. **a** Before NB generation (control); no particles are present in the DMEM. **b** After NB generation (case 1); a number of NBs (white dots) are detected. The difference in the size of the NBs can be attributed to the phase difference in the z-axis direction of the NBs in the DMEM. The NBs appear larger because they are scattered by the laser. The actual size of the NBs is determined using the NTA software with the captured video. **c** Size distribution and **d** concentration and mean diameter according to the results of the NB generation test, independently conducted five times (cases 1–5) to verify the reliability of the gas–liquid mixing method. The size of the generated NBs in the DMEM is polydisperse between 10 to 500 nm, and the mean diameter is confirmed to converge to less than 300 nm

**Fig. 2:**
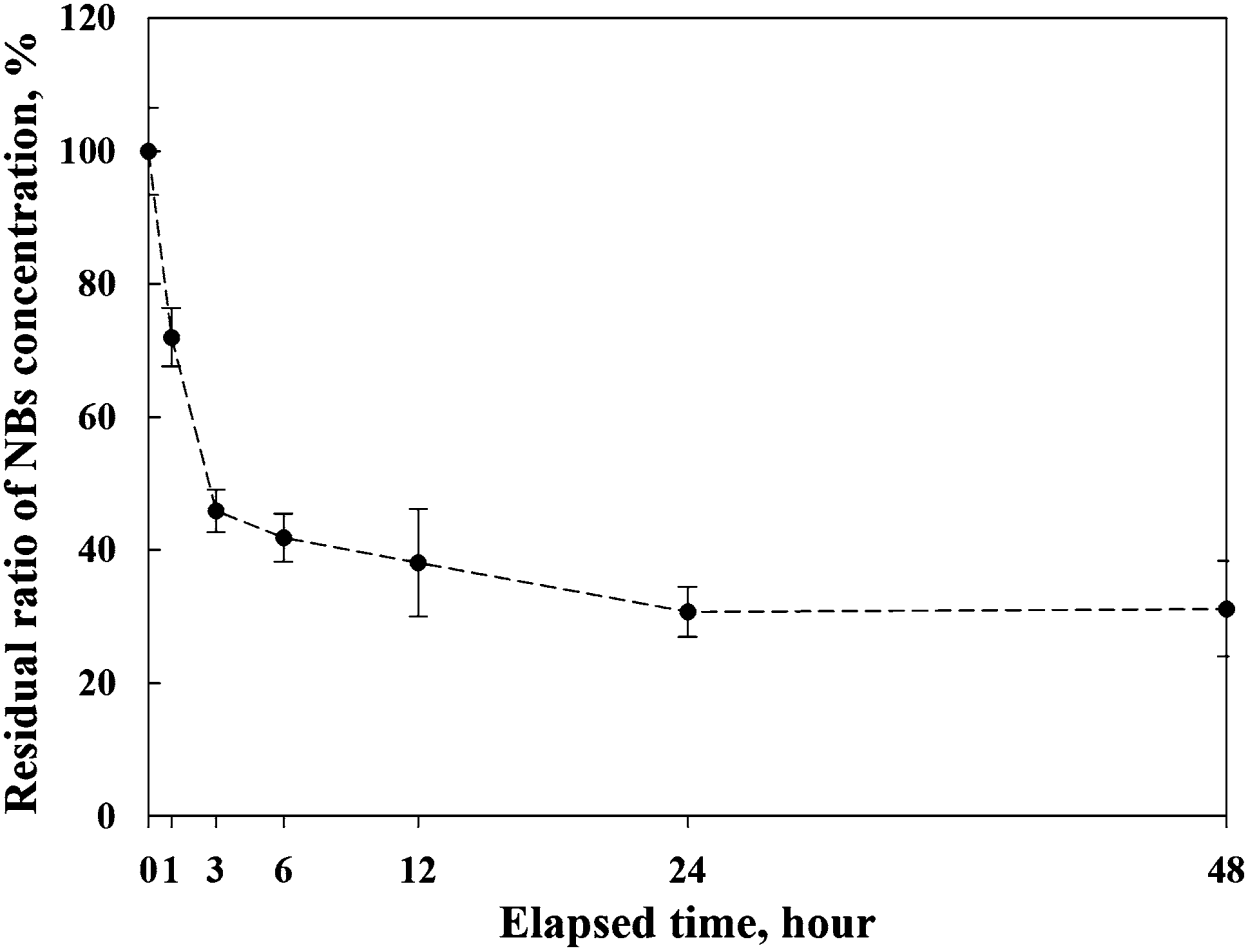
Residual ratio of the NB concentration over time in the DMEM. The concentration of the NBs in the DMEM decreased sharply for 3 h and later exhibited a gradual decrease to 31% of the initial concentration after 24 h. This value was maintained for the 48 h in which the cell culture was performed

The generated N-NBs in DMEM were examined, and their concentration and size were measured (Fig. 1). Figure 1a-b shows the images captured using a CCD camera with a NTA instrument. The black background and white dots represent DMEM and the generated NBs, respectively. In the DMEM without NBs (control), only a black background was observed (Fig. 1a). A large number of white dots (NBs) were observed in the DMEM containing N-NBs (Fig. 1b). Based on the analysis of the concentration and size of the generated N-NBs in DMEM (Fig. 1 c), polydispersed N-NBs were generated in the range of 10 to 500 nm. The concentration and average size of the N-NBs are shown in Fig. 1d. There was some variation in the number of N-NBs generated in DMEM; nevertheless, more than 400 million NBs were generated (case 1: 4.06 ± 0.15 × 10^8^ NBs/mL, case 2: 4.02 ± 0.71 × 10^8^ NBs/mL, case 3: 5.20 ± 0.76 × 10^8^ NBs/mL, case 4: 6.07 ± 0.74 × 10^8^ NBs/mL, case 5: 5.31 ± 0.50 × 10^8^ NBs/mL). Although size distribution of the N-NBs generated in DMEM using a gas–liquid mixing method showed some differences, each peak point of the graph (i.e., mode diameter) converged in the range of 100 ~ 300 nm. The change in the N-NB concentration over time is shown in Fig. 2. Overall, the N-NB concentration was decreased with time and was stably maintained at more than 31% after 48 h (1.71 ± 0.07 × 10^8^ NBs/mL). In general, the cause of the reduction in NBs may be flotation to the liquid surface or diffusion into the liquid. As the increasing velocity of the NBs is negligible, it is difficult to conclude that the NBs floated to the liquid surface and dissipated into the atmosphere. According to the Young–Laplace equation and Epstein-Plesset theory, NBs with a diameter of 200 nm should diffuse in liquid and disappear within 80 µs due to high internal pressure (i.e., pressure imbalance inside and outside the NBs) [29]. However, it was confirmed by attenuated total reflection (ATR-IR) analysis that the surface of NBs has tight hydrogen bonds [9]. NBs have been suggested to exist in a stable state because strong hydrogen bonds act as diffusion barriers and prevent the diffusion of the NBs. Ions adsorbed on the NB surface (i.e., model of ion-stabilized bubbles) could alleviate pressure imbalance inside and outside the NBs [30]. In addition, NBs may exist in a stable state because the hydrophobic material adsorbed on the NB surface maintains the balance between the inflow and outflow of gas (i.e., dynamic equilibrium model). Therefore, it is possible that 69% of the initially generated NBs diffused in DMEM and disappeared (unstable state), and 31% of bubbles, which were relatively stable, were maintained for 48 h. These results indicated that the concentration of the remaining N-NBs in the cell medium was sufficient to evaluate their effect on the cell culture.

### Cell population assay; NucBlue staining

**Fig. 3:**
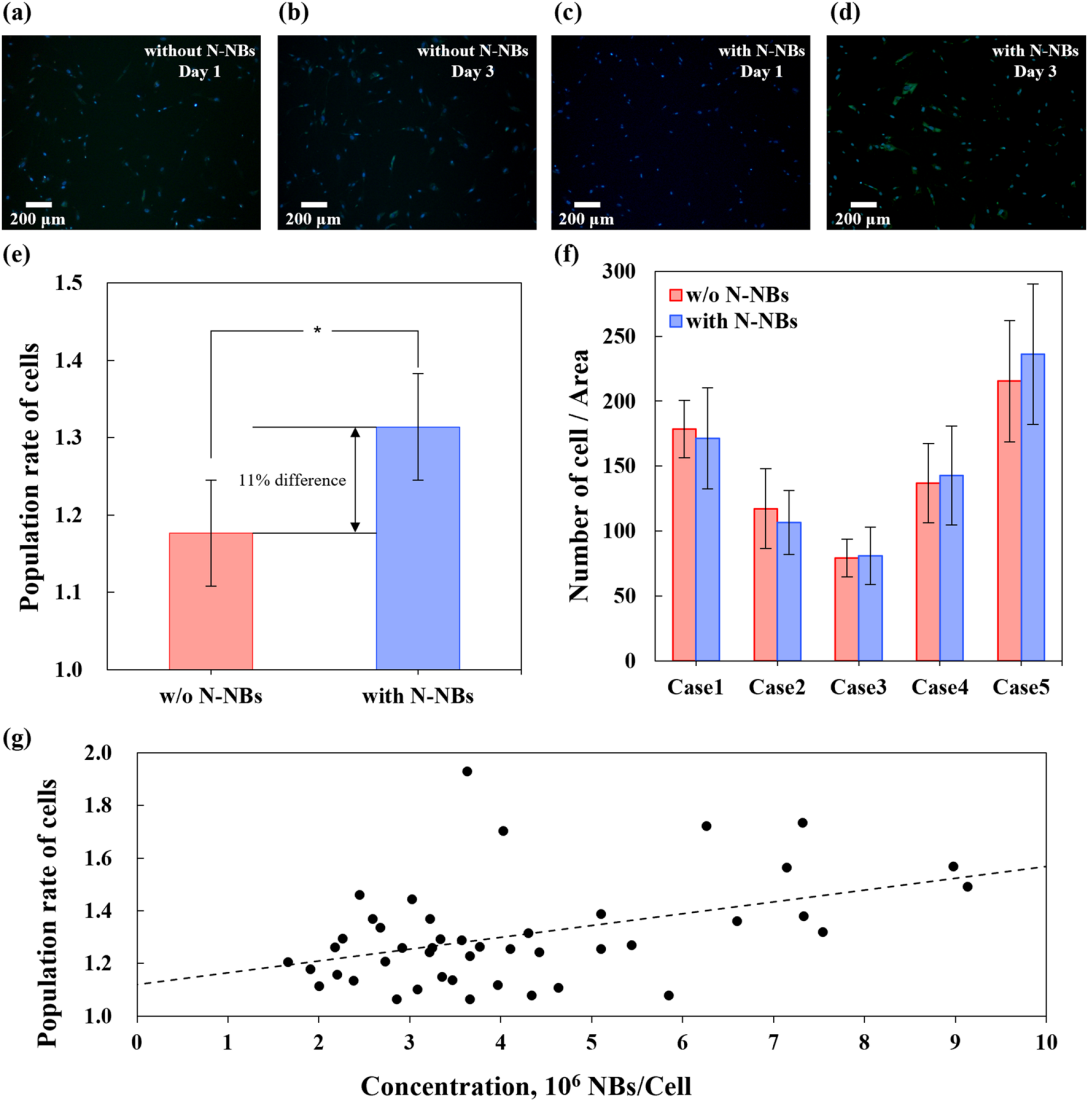
ImageJ-based image analysis of the MRC-5 cell nucleus stained with NucBlue. **a** Fluorescence image without N-NBs on day 1. **b** Fluorescence image without N-NBs on day 3. **c** Fluorescence image with N-NBs on day 1. **d** Fluorescence image with N-NBs on day 3. **e** Population rate of cells between days 1 and 3. **f** Difference in the number of cells on day 1 for the different cases. The number of cells attached to the bottom varies among the cases. **g** Population rate of cells normalized by the number of NB density per cell (concentration). The Population rate of cells increases with NB density

To evaluate the effect of N-NBs on cells, increase in cell number was examined by image analysis using ImageJ. Fluorescence image on day 1 and day 3 with N-NBs and without N-NBs are shown in Fig. 3a-d. The fluorescence image (Fig. S1 (a)) was converted to a black and white image, and the brightness was adjusted so that the nucleus of the cells was not affected to minimize noise (Fig. S1 (b)). The stained cell nucleus and noise (bright region of the fluorescence image) were converted to white dots. To remove noise, white dots with a diameter of 5 pixels or less were removed (Fig. S1 (c)). White dots with an area of 10 pixels^2^ or more were counted (Fig. S1 (d)). The results of cell counting using ImageJ showed that in the control group, increase in cell number was increased on average of 1.18 times between day 1 and day 3, and in the experimental group, the average value was 1.31 times, which was 11% higher than that in the control group (Fig. 3e). Although the cells were seeded at a density of 5,000 cells/mL, there was a difference in the cells attached to the bottom of the well (Fig. 3 (f)), and the number of NBs generated in DMEM was also different within a certain range (Fig. 1d). Considering the influence of cell attachment and NB concentration, the normalization method was used. The number of NBs (N) was divided by the number of cells normally attached to the floor on day 1 (M) to normalize as the number of NBs per cell (K = N/M), and the increase in cell number was evaluated. The results showed that increase in cell number of cells tended to be proportional to the number of NBs per cell (K). The trend can be expressed with a linear equation: *y* = 4.48 × 10^–8^ x + 1.12 (Fig. 3g). The slope was very small, *y* = 4.48 × 10^–8^; that means, a weak association was observed between the number of N-NBs per cell and the cell number increase rate. In statistical analysis using SPSS Statistics (IBM Co., USA), a statistical significance was observed in increase in cell number (Fig. 3e). Although there was no large difference in the increase in cell number for 3 days, the effect of N-NBs on increase in cell number could be greater in a long-term culture environment for 1 week or longer.

### Cell division and cycle assays

**Fig. 4:**
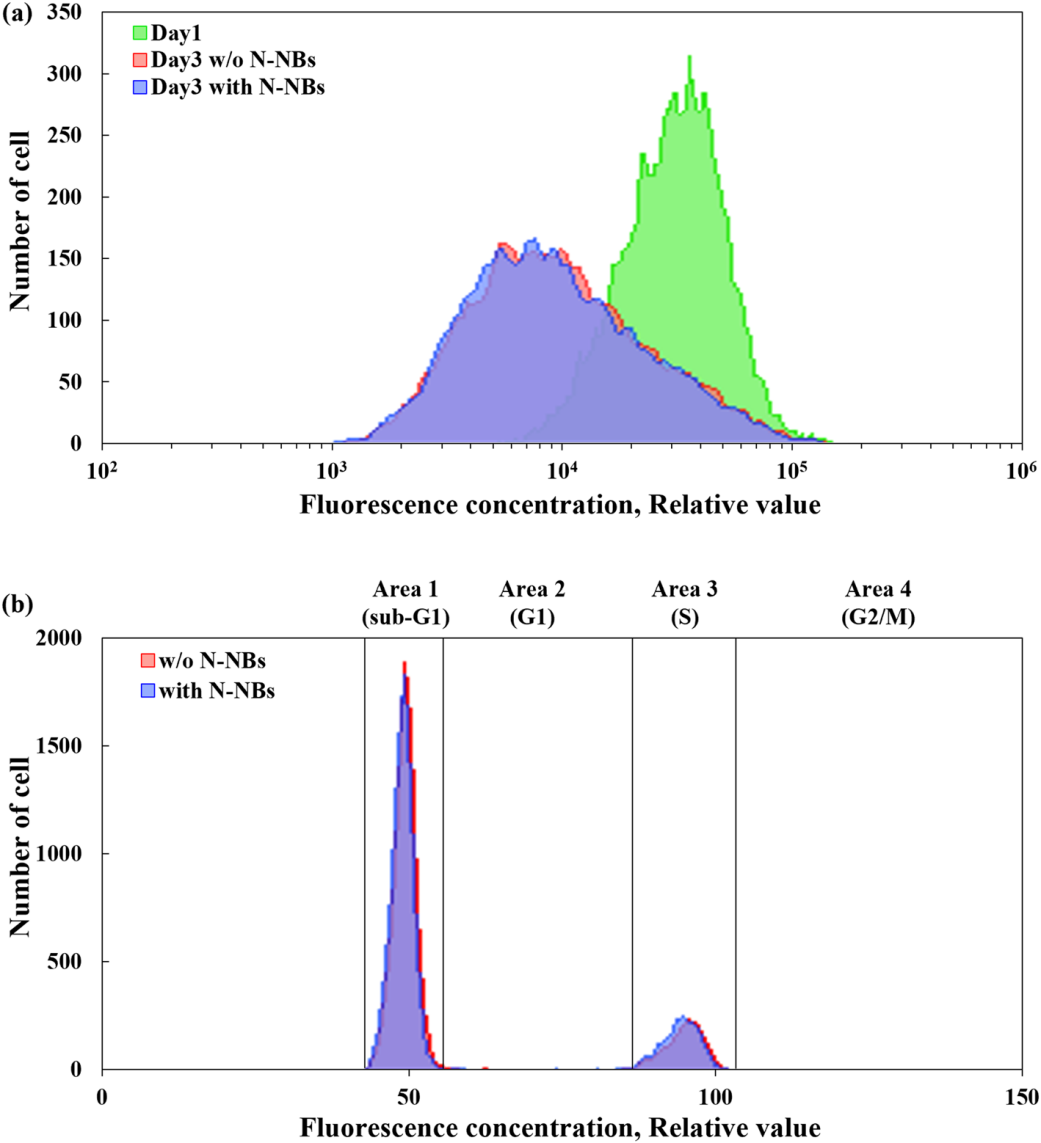
**a** CFSE analysis data. On day 1, the mean fluorescence intensity is 32,828. On day 3, the average fluorescence intensity is 14,427 and 14,156 in the culture medium without N-NBs and with N-NBs, respectively. A lower mean fluorescence concentration corresponds to a larger number of proliferated cells. **b** PI-RNase analysis data. The histogram shows the distribution of cells in each area in the culture medium with and without N-NBs. Area 1 (sub-G1 phase) includes dead cells with less than n chromosomes. In Area 2 (G1 phase), cells have 2n chromosomes and are prepared for chromosome replication. In Area 3 (S phase), the cells replicate the chromosomes and have 2n–4n chromosomes. In Area 4 (G2/M phase), the cell is divided in two and has 4n chromosomes. As a result, in the two assays, the difference between the culture medium with N-NBs and the culture medium without N-NBs is small, so it is difficult to say that it is significant

It was inferred that the differences in cell numbers were due to cell proliferation, and two additional analyses were conducted to find further evidence of the effect of NBs on cell proliferation. The first analysis was CFSE (Carboxyfluorescein Succinimidyl Ester) staining. CFSE stains the cell nucleus, and the fluorescence intensity is reduced by half per division of cell. Therefore, it is possible to determine the number of proliferating cells based on the fluorescence intensity. In the control group and the experimental group, the fluorescence intensity on day 3 (the degree of shifting to the left side of the graph) was compared with the fluorescence intensity on day 1. The results showed that the average CFSE fluorescence intensity of the control group and the experimental group on day 3 was decreased from 32,828 to 14,427 and 14,156, respectively (Fig. 4a). The CFSE fluorescence intensity was lower by 1.9% in the experimental group compared with the control group. Additionally, PI-RNase (Propidium Iodide-Ribonuclease) staining was performed to measure the difference in the number of cells according to the phase of the cell cycle. Since cell proliferation is closely related to the cell cycle, this method is reasonable. PI-RNase staining is analyzed by flow cytometry (Fig. 4b). Area 1 is the sub-G1 phase with dead cells, and the cells have less than 2n chromosomes (Fig. 4b). Area 2 is the G1 phase in which cells with 2n chromosomes prepare for replication. Area 3 is the S phase in which chromosomes replicate and become chromatids; in this step, the number of chromosomes increases from 2 to 4n. Lastly, area 4 is the G2/M phase in which the chromatids are ready for separation by the kinetochore microtubules, and the nucleus and cytoplasm are divided; in this phase, the number of chromosomes is 4n. A comparison of the population rate of cells in each phase between the control group and experimental group revealed that it was 1.93% lower in the G1 phase and 0.42% and 1.57% higher in the S phase and G2/M phase, respectively, in the experimental group (Fig. S2).

In these two assays (CFSE staining and PI-RNase staining), the differences in cell proliferation in culture medium with and without N-NBs was relatively small. Therefore, we should not rush to conclude that cell division is the cause of the significant increase in cell numbers in culture media containing N-NBs. However, two additional potential issues need to be considered. The first is the fact that small differences of 1.9% and 1.93%, respectively, in CFSE and PI-RNase assays can be accumulated, resulting in large differences. The other is the possibility that N-NBs may have a stronger effect on cell metabolism and apoptosis mechanism. It is important to understand that issues like these need to be studied in more detail.

## Conclusions

In this study, the proliferative effect of N-NBs on MRC-5 cells was investigated. For MRC-5 cell culture, N-NBs were generated in DMEM using a gas–liquid mixing method at a high concentration (more than 4 × 10^8^ NBs/mL). In addition, the N-NBs were stably maintained at more than 31% (1.71 ± 0.07 × 10^8^ NBs/mL) after 48 h. To evaluate increase in cell numbers, image analysis with NucBlue staining was performed. The results of image analysis and flow cytometry revealed that proliferation in the culture medium with N-NBs was increased by 11%, compared with that in the culture medium without N-NBs. We assessed the cell cycle via CFSE and PI-RNase, speculating that differences in cell numbers were caused by cell proliferation. The result indicated that the cell proliferation in the culture medium with and without N-NBs was not significantly different (1.9% and 1.93% respectively). Although our hypothesis was wrong, the image analysis showed that the cells cultured in the culture medium with N-NBs retained more cells. This means that the N-NBs in culture medium has a clear effect on the cells. Thus, there is a need to study the effect of culture medium with N-NBs on cells through more diverse assays. Furthermore, studies on the effects of NBs with various types of gas on cell culture will be required for a more comprehensive understanding of the biological interactions between NBs and cells. To the best of our knowledge, this study is the first to investigate the effect of N-NBs (directly generated in DMEM) on a fibroblast cell culture. Our study could serve as a reference for future studies on the biological effect of NBs on living organisms.

## Supplementary Information

Below is the link to the electronic supplementary material.

Supplementary file1 (DOCX 2598 KB)
