# Supplementary material for: Proliferative effects of nanobubbles on fibroblasts
Source: Biomed Eng Lett. 2022 Aug 13;12(4):393–400. doi: 10.1007/s13534-022-00242-y (PMC9550906; doi:10.1007/s13534-022-00242-y)
Supplement: Supplementary file 1 — Supplementary file1 (DOCX 2598 KB) [file 13534_2022_242_MOESM1_ESM.docx]

**Supplementary Material**

**Proliferative effects of nanobubbles on fibroblasts**

**Hansol Heo^1^, Junseon Park^1^, Jeong Il Lee^1,2^, Jungho Kim^3^, Joong Yull Park^1,4^* and Jong-Min Kim^1^***

^1^School of Mechanical Engineering, Chung-Ang University, Seoul 06974, Republic of Korea

^2^Department of Computer Science and Engineering, Chung-Ang University, Seoul 156-756, Republic of Korea

^3^Department of Life Science, Sogang University, Seoul 04107, Republic of Korea

^4^Department of Intelligent Energy and Industry, Chung-Ang University, Seoul 06974, Republic of Korea


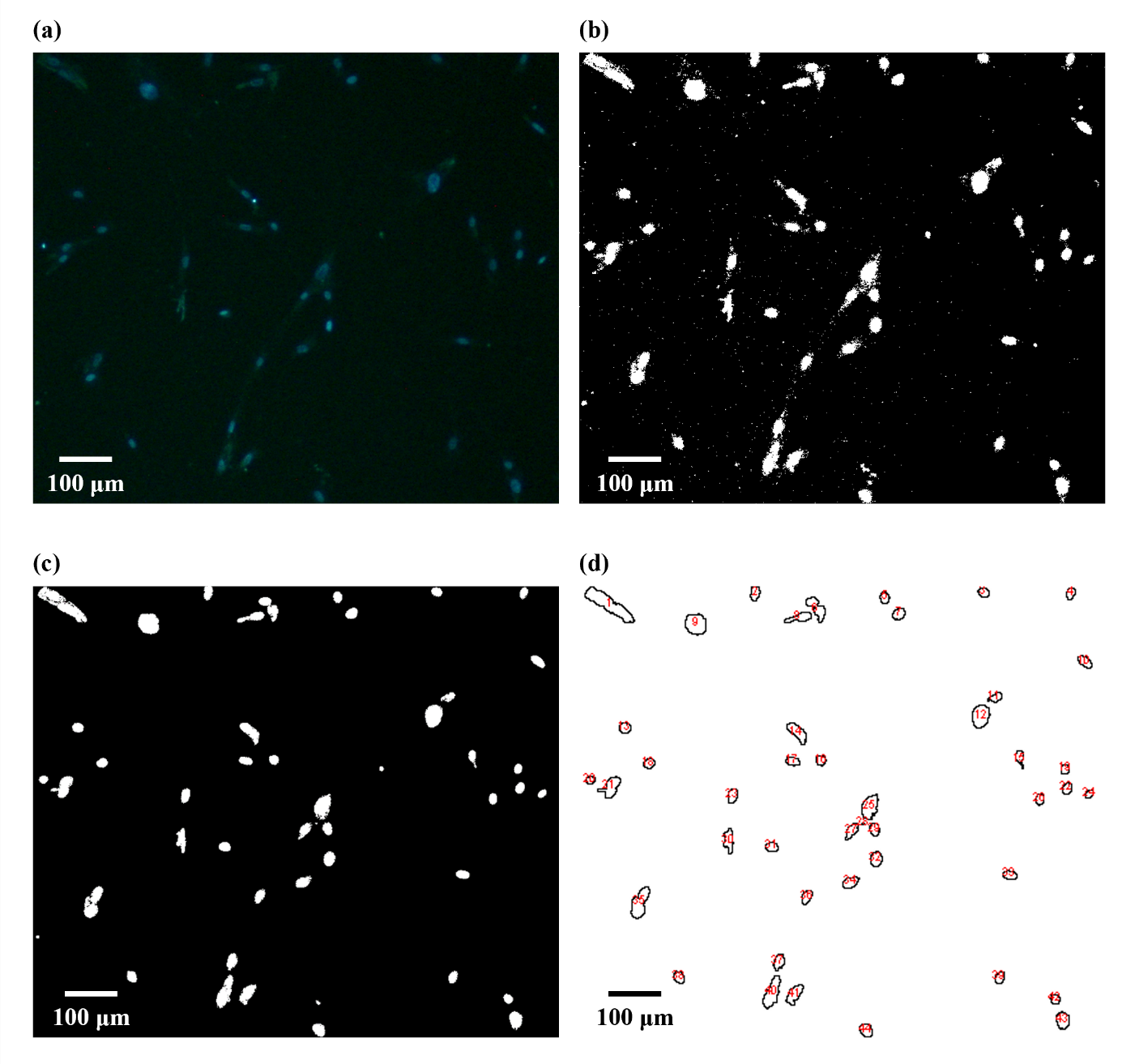


**Fig. S1**. Image analysis method for the MRC-5 cell nucleus stained with NucBlue using ImageJ. (a) Original fluorescence image. (b) Simplified black and white image. (c) Image with noise removed. (d) Quantification of the number of cells.


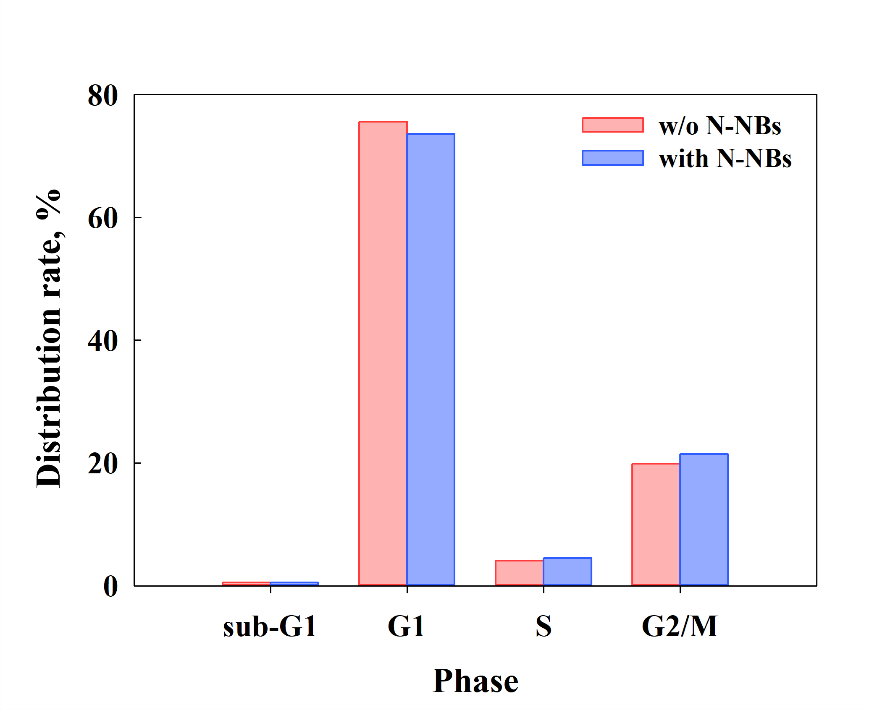


**Fig. S2**. Cell distribution rate in each phase. Compared with that in the culture medium without N-NBs, the population of cells in the G1 phase in the culture medium with N-NBs is smaller and that in the S phase and G2/M phase is larger. The total number of cells is 10,000.
